# The study of honokiol as a natural product-based antimicrobial agent and its potential interaction with FtsZ protein

**DOI:** 10.3389/fmicb.2024.1361508

**Published:** 2024-07-22

**Authors:** Ning Sun, Ziling Zhi, Ting Xiao, Xin Deng, Tenghui He, Wanyang Dong, Shuyi Feng, Sisi Chen, Wing-Leung Wong, Wenchang Yuan

**Affiliations:** ^1^Guangzhou 11th People's Hospital, Guangzhou Cadre and Talent Health Management Center, Guangzhou, China; ^2^Guangzhou Key Laboratory for Clinical Rapid Diagnosis and Early Warning of Infectious Diseases, King Med School of Laboratory Medicine, Guangzhou Medical University, Guangzhou, China; ^3^State Key Laboratory of Chemical Biology and Drug Discovery, and Department of Applied Biology and Chemical Technology, The Hong Kong Polytechnic University, Kowloon, Hong Kong SAR, China

**Keywords:** bacterial resistance, antibacterial activity, honokiol, cell division, FtsZ inhibitor

## Abstract

Multidrug resistant bacteria have been a global health threat currently and frontline clinical treatments for these infections are very limited. To develop potent antibacterial agents with new bactericidal mechanisms is thus needed urgently to address this critical antibiotic resistance challenge. Natural products are a treasure of small molecules with high bioactive and low toxicity. In the present study, we demonstrated that a natural compound, honokiol, showed potent antibacterial activity against a number of Gram-positive bacteria including MRSA and VRE. Moreover, honokiol in combination with clinically used β-lactam antibiotics exhibits strong synergistic antimicrobial effects against drug-resistant *S. aureus* strains. Biochemical studies further reveal that honokiol may disrupt the GTPase activity, FtsZ polymerization, cell division. These biological impacts induced by honokiol may ultimately cause bacterial cell death. The *in vivo* antibacterial activity of honokiol against *S. aureus* infection was also verified with a biological model of *G. mellonella* larvae. The *in vivo* results support that honokiol is low toxic against the larvae and effectively increases the survival rate of the larvae infected with *S. aureus*. These findings demonstrate the potential of honokiol for further structural advancement as a new class of antibacterial agents with high potency against multidrug-resistant bacteria.

## 1 Introduction

Over the past decades, antibiotics have been widely utilized to treat various bacterial infections and protected the global public health. However, due to the inappropriate administration of antibiotics, bacteria have developed strong defense mechanisms against the conventional antibiotics and that results in drug resistance (Aminov, 2021; Smith et al., 2023). At present, antimicrobial resistance stands as a critical and worldwide predicament. Notable examples of antibiotic resistant bacteria include vancomycin-resistant *Enterococcus faecium* (VREF) and methicillin-resistant *Staphylococcus aureus* (MRSA). Both emblematic of bacteria are resistant to conventional antibiotics such as vancomycin and methicillin (Simonetti et al., 2022; Cimen et al., 2023). Given the rapid emergence of drug-resistant bacteria as well as the dwindling options for clinical treatments, an urgent is thus needed to develop alternative strategies to combat drug-resistant bacterial infections.

**Figure 1:**
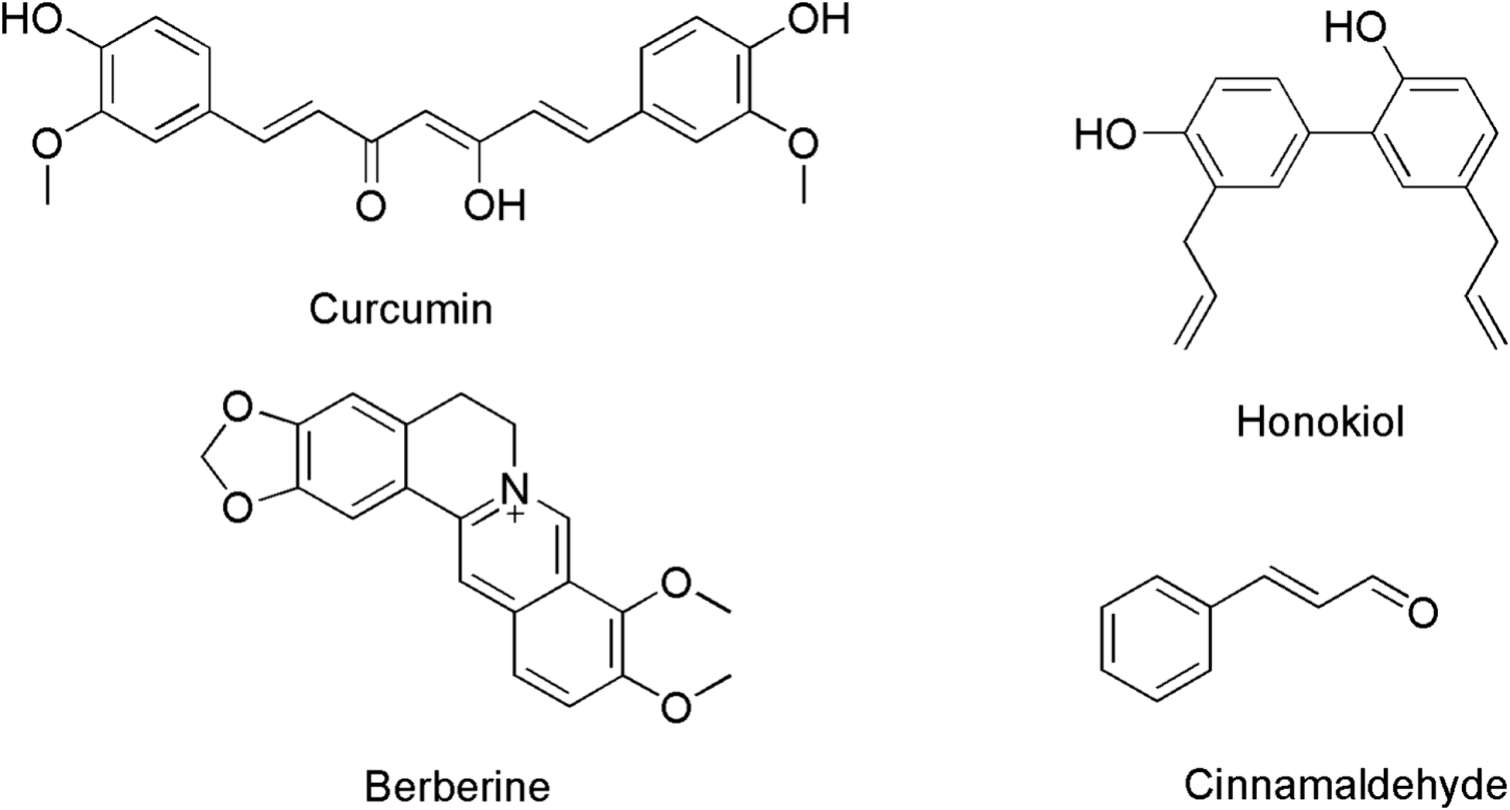
Chemical structures of curcumin, berberine, cinnamaldehyde and honokiol.

Throughout the history of antibiotic discovery, natural products have played a pivotal role, including the production of widely utilized antibiotics like penicillin and vancomycin. Today, there is ongoing anticipation that natural products may yet again provide solutions to the antibiotic crisis that we face (Wright, 2017). Honokiol (Figure 1) is an herbal chemical component originating from Asia, primarily found in the bark and flowers of *Magnolia officinalis* and *Styrax obassia* trees. These plants have a long history in traditional Chinese medicine and have been employed to treat certain diseases or symptoms such as asthma, abdominal discomfort and pain, indigestion, and cough associated with asthma (Rauf et al., 2021). In recent years, an increasing number of studies have shown that honokiol may possess a wide range of pharmacological effects, such as anticancer, antioxidant, anti-inflammatory, and antiviral properties (Rauf et al., 2021). Honokiol is considered safety to human body (Sarrica et al., 2018) and its inhibitory effects on *Nocardia seriolae* has also been reported previously (Jiang et al., 2022). However, the antibacterial mechanism of honokiol is still not clear. In this study, we evaluated the antimicrobial activity of honokiol against a panel of bacteria and attempted to understand its potential interactions with the purified bacterial Filamenting temperature-sensitive mutant Z (FtsZ) that has been known taking vital role in bacterial cell division.

Regarding recent advancements in the discovery of new drugs, the bacterial divisome has emerged as a promising drug target for the development of the next generation of antibiotics. Within the divisome, particular attention has been given to FtsZ, recognized as a pivotal component (Casiraghi et al., 2020; Silber et al., 2020). In the process of bacterial cell division, FtsZ undergoes assembly to form a circular structure known as Z-ring at the division site. The process of cell division involves the binding and hydrolysis of GTP. Subsequently, FtsZ becomes stabilized and affixed to the inner surface of the cytoplasmic membrane through interactions with FtsZ-binding proteins. Once the Z-ring is established, FtsZ orchestrates the recruitment of various other proteins responsible for initiating cytokinesis (Bisson-Filho et al., 2017; Yang et al., 2017; Monteiro et al., 2018). The substantial conservation and functional significance of FtsZ render it an attractive target for the development of innovative antibacterial agents (Haranahalli et al., 2016; Hurley et al., 2016). Recently, several natural products have been reported as FtsZ-targeting compounds, such as berberine, curcumin and cinnamaldehyde (Figure 1). Bacterial cell division was found disrupted by inhibiting FtsZ activity with these compounds (Domadia et al., 2007, 2008; Rai et al., 2008). In attempts to search for the potential targets of honokiol in bacteria, we observe that honokiol may interrupt the Z-ring formation and cell division on *B. subtilis* cells. We therefore performed experiments to understand the interaction of honokiol FtsZ as such interactions that could inhibit the proper function of FtsZ in bacterial cells.

## 2 Results and discussion

### 2.1 *In vitro* antibacterial activity of honokiol

To evaluate the antibacterial activity of honokiol, a number of bacteria including drug-resistant strains were utilized in the present study. Methicillin and berberine were also investigated under the same conditions for comparison. The minimum inhibitory concentrations (MIC) values obtained are summarized in Table 1. The results showed that honokiol could inhibit the growth of all Gram-positive strains examined and the MIC values were found ranging from 4 to 12 μg/mL. Honokiol exhibited comparable inhibitory effects against *B. subtilis* 168, *S. aureus* ATCC 29213, and *S. epidermidis* ATCC 12228 with MIC at 4 μg/mL, which was notably better than berberine. Furthermore, honokiol displayed remarkable antibacterial activity against methicillin-resistant *S. aureus* (MRSA), including strains ATCC BAA-41, 33591, 33592, *et cetera*, with MICs ranging from 4 to 8 μg/mL. The antibacterial activity of honokiol was found 100-fold better than that of methicillin and berberine. Honokiol also exhibited a robust inhibitory effect on the growth of *E. faecalis* and *E. faecium*, with an MIC of 8 μg/mL. Notably, vancomycin against vancomycin-resistant *E. faecalis* and *E. faecium* (VREs) exhibited high MICs (> 96 μg/mL). The results indicate the superior antibacterial activity of honokiol against VREs as compared to vancomycin (Sun et al., 2014). However, honokiol even at 256 μg/mL exhibited no antibacterial activity against Gram-negative bacteria such as *E. coli*, *P. aeruginosa*, and *K. pneumoniae*. One of the possible reasons may be attributed to the low penetration ability of honokiol against out membrane of Gram-negative strains.

**Table 1:** Antibacterial activity of honokiol against a panel of bacterial strains.

Bacterial strains	MIC (μg/mL)		
	HNK	MET	BER
*B. subtilis* 168	4	<1	96
*S. aureus* ATCC 29213	4	1	192
*S. aureus* ATCC BAA-41^a^	4	1,024	192
*S. aureus* ATCC BAA-1717^a^	4	256	192
*S. aureus* ATCC BAA-1720^a^	4	256	192
*S. aureus* ATCC 33591^a^	8	1,024	192
*S. aureus* ATCC 33592^a^	8	512	192
*S. aureus* ATCC 43300^a^	4	512	192
*S. aureus* ATCC 29247^b^	4	6	192
*S. epidermidis* ATCC 12228	4	0.75	192
*E. faecalis* ATCC 29212	12	4	>192
*E. faecalis* ATCC 51575^c^	12	4	>192
*E. faecium* ATCC 49624	12	4	>192
*E. faecium* ATCC 700221^c^	12	4	>192
*E. coli* ATCC 25922	>256	4	>192
*P. aeruginosa* ATCC BAA-2108	>256	>1,024	>192
*K. pneumoniae* ATCC BAA-1144	>256	>1,024	>192

a MRSA.

b Ampicillin-resistant strain.

c Vancomycin-resistant strains.

HNK, honokiol; MET, methicillin; BER, berberine.

### 2.2 Time-killing curve determinations of honokiol

**Figure 2:**
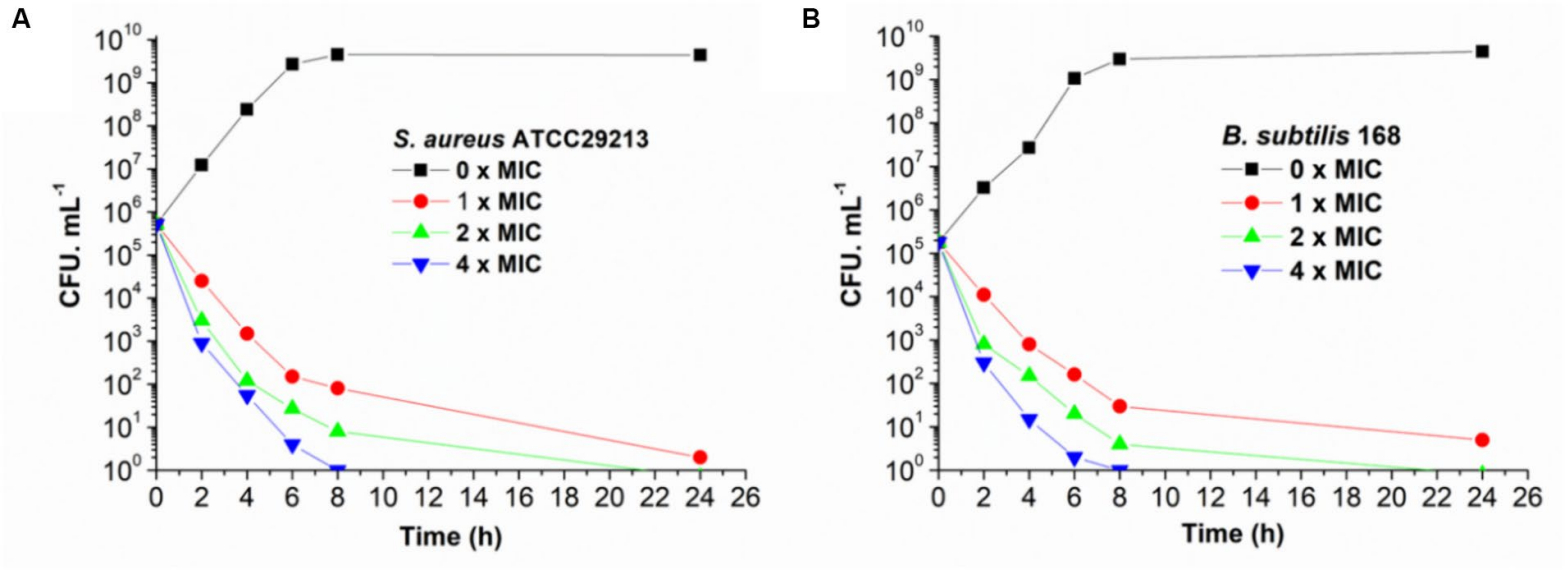
Time-killing curve of honokiol against *S. aureus* ATCC 29213 **(A)** and *B. subtilis* 168 **(B)**. Different concentrations of honokiol were represented by different colors.

The bactericidal and bacteriostatic nature of honokiol against bacteria were investigated and the viable counts were conducted following established protocols (Wikler et al., 2009). Killing curves obtained from the action of honokiol against *B. subtilis* 168 and *S. aureus* ATCC 29213 are depicted in Figure 2. The control group exhibited rapid growth in CFU counts as compared to the initial inoculum. In Figure 2A, the results show that honokiol at 1 × MIC causes a reduction of 1 × 10^2^ CFU/mL against *S. aureus* within 6 h. Moreover, the viable count of bacteria was found below the lowest detectable limit (10^3^ CFU/mL) within 24 h. In the *B. subtilis* bacterial survival assays (Figure 2B), honokiol at 4 × MIC lowered the viable counts rapidly below the lowest detectable limit after 4 h of incubation, and maintained the viable counts under the detectable limit for over 24 h at MIC concentration. We also obtained the time-killing curves of honokiol against drug-resistant strains, including *S. aureus* ATCC 33591 and ATCC 43300, and *E. faecium* ATCC 700221. The observation (Supplementary Figure S1) was found similar to those observed in *B. subtilis* 168 and *S. aureus* ATCC 29213. These results may indicate that the antibacterial activity of honokiol aligns with a bactericidal mode of action.

### 2.3 Synergistic effects of honokiol with β-lactam antibiotics

**Figure 3:**
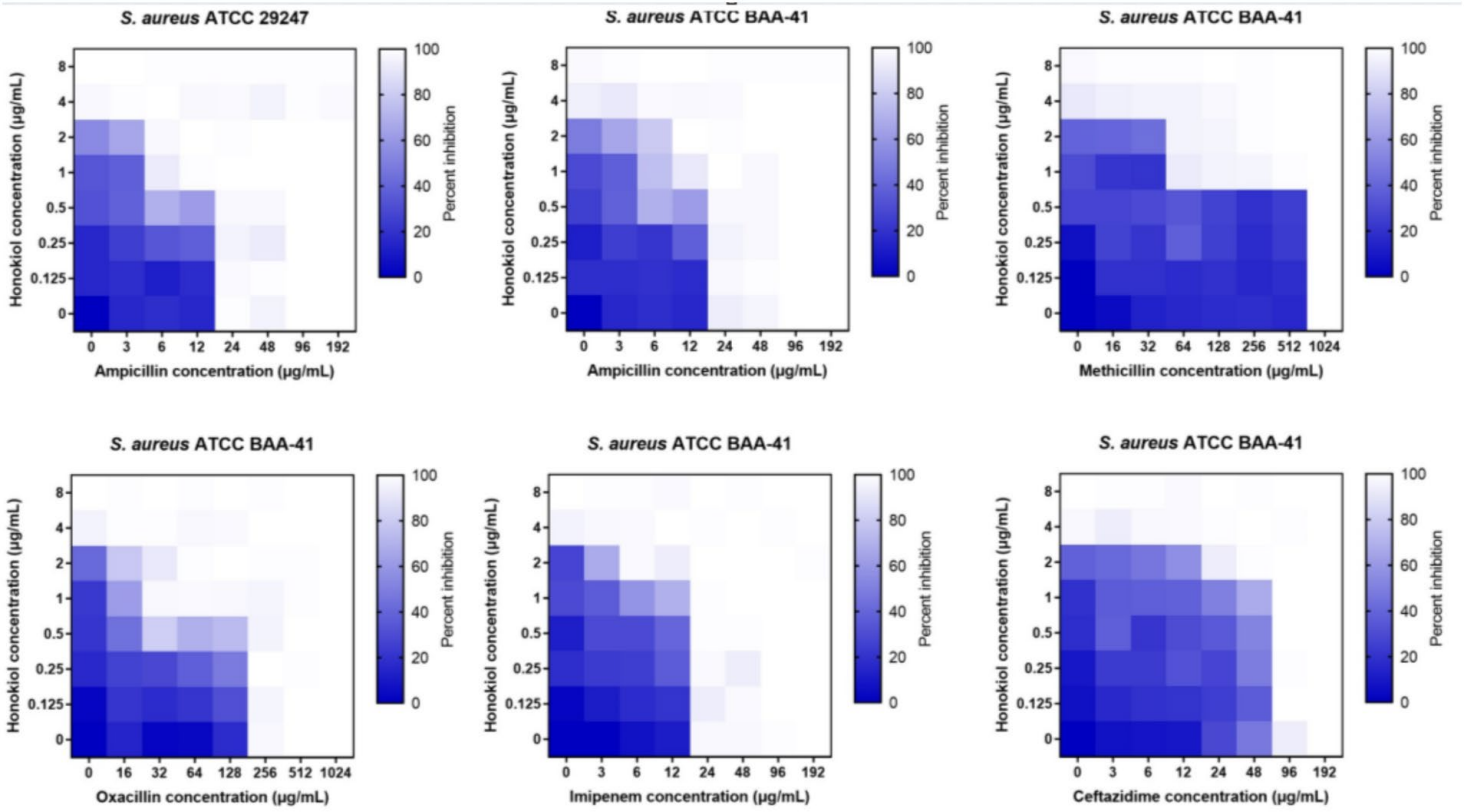
Synergistic effects of honokiol with β-lactam antibiotics against drug-resistant *S. aureus.*

To evaluate the potential of honokiol in restoring the antibacterial efficacy of β-lactam antibiotics against ampicillin-resistant *S. aureus* and MRSA (ATCC BAA-41), a broth microdilution checkerboard experiment was conducted. From Table 2 and Figure 3, the results illustrate that β-lactam antibiotics display modest or moderate antibacterial activity against drug-resistant *S. aureus*, with MIC values exceeding 24 μg/mL. In general, the combination of these β-lactam antibiotics with honokiol is able to enhances the antibacterial activity effectively against the strains tested. Honokiol at 1 μg/mL enhanced the antibacterial effectiveness of ampicillin against ampicillin-resistant *S. aureus*, as indicated by the reduced MIC value of ampicillin from 24 μg/mL to 6 μg/mL. Furthermore, honokiol at 1 μg/mL significantly improved the bacteria-killing activity of methicillin against MRSA (ATCC BAA-41) and lowered the MIC value from 1,024 μg/mL to 64 μg/mL. A Fractional Inhibitory Concentration Index (FICI) observed was 0.3125.

**Table 2:** MIC values of honokiol combined with β-lactam antibiotics against drug-resistant *S. aureus*.

Strain^a^	Compound	MIC (Single compound, μg/mL)	MIC (Checkerboard assay, μg/mL)	FICI
ATCC 29247	Honokiol	4	1	0.5
	Ampicillin	24	6	
ATCC BAA-41	Honokiol	4	1	0.5
	Ampicillin	48	12	
ATCC BAA-41	Honokiol	4	1	0.3125
	Methicillin	1,024	64	
ATCC BAA-41	Honokiol	4	1	0.375
	Oxacillin	256	32	
ATCC BAA-41	Honokiol	4	2	0.75
	Imipenem	24	6	
ATCC BAA-41	Honokiol	4	2	0.75
	Ceftazidime	96	24	

a ATCC 29247 is an ampicillin-resistant *S. aureus*. ATCC BAA-41 is an MRSA.

The combination of honokiol with oxacillin or ampicillin also exhibited synergistic effects against BAA-41 strain, with FICIs of 0.5 and 0.375, respectively. In these assays, honokiol at 1 μg/mL enhanced the bacteria-killing potency of ampicillin by 4 folds (MIC was reduced from 48 μg/mL to 12 μg/mL) and oxacillin by 8 folds (MIC was reduced from 256 μg/mL to 32 μg/mL) against BAA-41 strain. A partial synergistic effect with FICIs of 0.75 for the combination of honokiol with imipenem or ceftazidime were also observed in the antibacterial study.

### 2.4 The antibacterial effect of honokiol in combination with PMBN against gram-negative strains

The cell wall of Gram-negative bacteria is different from that of Gram-positive bacteria, possessing special components such as lipopolysaccharides, outer membrane, and lipoproteins in addition to the peptidoglycan layer. Some antimicrobial substances, such as penicillin and trypsin, have poor antibacterial effects on Gram-negative bacteria due to the bacterial outer membrane (Smith et al., 2023). PMBN (polymyxin B nonapeptide), capable of improving the permeability of the outer membrane of Gram-negative bacteria (Tsubery et al., 2000), was utilized to improve the antibacterial effect of honokiol against *E. coli* and *K. pneumoniae* by enhancing the permeability of the compound. Table 3 displays the antimicrobial activity of honokiol in combination with PMBN. The results indicate that neither PMBN nor honokiol at 32 μg/mL exhibits observable antibacterial effects against the strains tested. Nevertheless, when honokiol combining with PMBN at 20 μg/mL, it showed antibacterial activity against both *K. pneumoniae* and *E. coli* and a MIC value of 32 μg/mL was obtained.

**Table 3:** Antimicrobial activities of honokiol and PMBN against *E. coli* and *K. pneumoniae* (The concentration of PMBN in the combination test is fixed at 20 μg/mL).

Compounds	MIC (μg/mL)			
	*E. coli* ATCC 25922		*K. pneumoniae* ATCC BAA-1144	
	−PMBN	+PMBN	−PMBN	+PMBN
PMBN	>32	–	>32	–
Honokiol	>32	32	>32	32

### 2.5 The effect of honokiol on bacterial cell morphology and membrane of *Bacillus subtilis*

**Figure 4:**
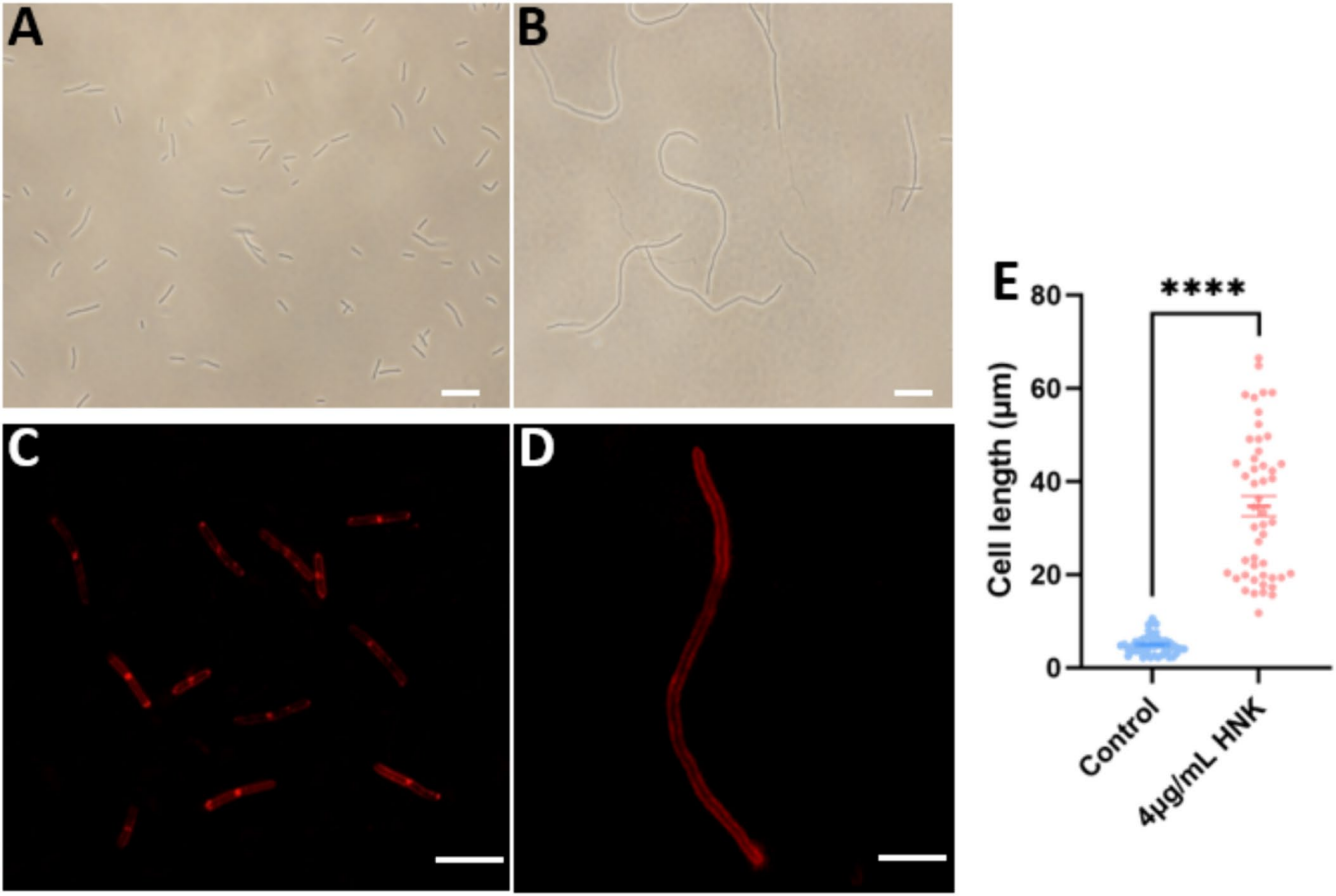
The effect of honokiol on bacterial morphology and membrane of *B. subtilis*. The *B. subtilis* cells were grown in the absence **(A)**, and in the presence **(B)** of 4 μg/mL of honokiol. Observations after membrane staining with the red fluorescent dye FM 4–64 are shown in the absence **(C)** or in the presence **(D)** of 4 μg/mL of honokiol (The scale bar is 10 μm). **(E)** Comparison of the cell length distributions of the *B. subtilis* cells (the number of the cell is 50 for each group) treated by 1%(v/v) DMSO and the 4 μg/mL of honokiol. ****The result has statistical significance.

FtsZ-targeting compounds are known to exert antibacterial function by inhibiting FtsZ activity and inducing cell elongation (Hufford and Lasswell, 1978; Rai et al., 2008; Ruiz-Avila et al., 2013). To get more understanding on the possible antibacterial mechanism of honokiol, we investigated its effects on *B. subtilis* cell division. The morphology of *B. subtilis* cells exposed to the conditions with and without honokiol was examined with microscopy techniques. The results revealed a significant elongation of *B. subtilis* cells in the presence of honokiol as compared with the control. Under normal conditions, ~60% of *B. subtilis* cells are found 3–5 μm in length, and ~ 38% are found in 5–10 μm. Only ~1.5% of the cells are found in 10–15 μm. The average length of the cells is 4.6 ± 0.29 μm (Figures 4A,E). However, most of the cells treated with honokiol at the MIC concentration (4 μg/mL) exhibited a length exceeding 20 μm, and ~ 45% of the cells are longer than 30 μm. And the average cell length is 34.7 ± 2.20 μm (Figures 4B,E). This phenomenon of cell division inhibition found is the same as reported FtsZ inhibitors such as TXA707, pyrimidine and quinoline derivatives (Fang et al., 2019a; Li et al., 2020; Ferrer-Gonzalez et al., 2021). Since disturbances to the bacterial cell membrane could trigger lysis and demise, we thus further investigated whether honokiol affected the integrity of the bacterial membrane. This was accomplished by incorporating FM4-64, a red fluorescent dye, to examine the membrane’s response to honokiol. As shown in Figure 4D, although *B. subtilis* cells exhibited an elongated morphology, honokiol did not cause any measurable disturbance of the cell membrane, as indicated by the high similarity between the treated and untreated cells in Figure 4C. In addition, no septum was observed in the elongated cells (Figure 4D). These results suggest that honokiol could inhibit the growth of *B. subtilis* by promoting the inhibition of cell division without compromising the bacterial membrane. These results prompted us to conduct further investigations into the underlying antibacterial mechanism of honokiol.

### 2.6 The effect of honokiol on FtsZ activity *in vitro*

**Figure 5:**
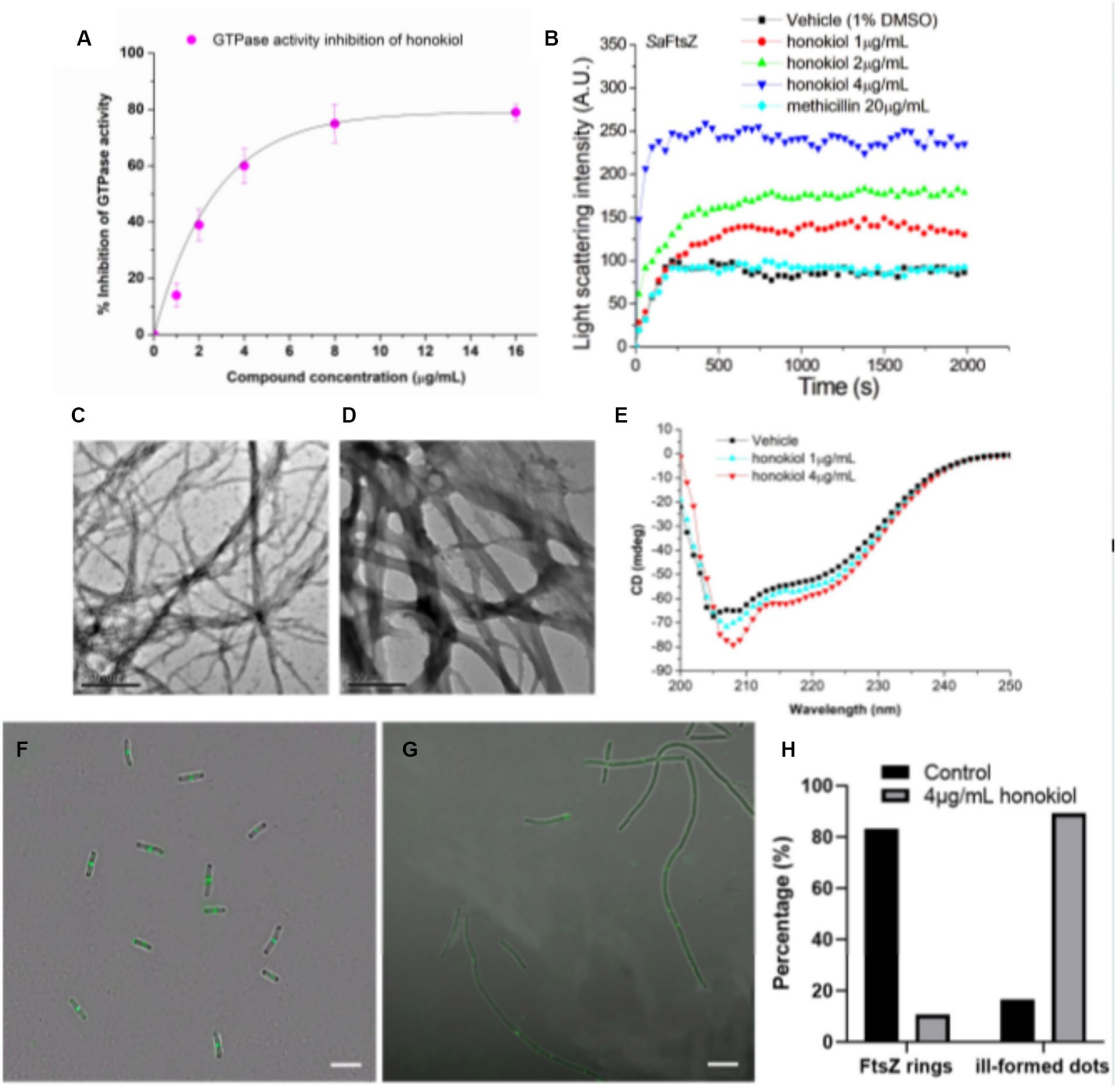
Effects of honokiol on the FtsZ. **(A)** Inhibition of GTPase activity of FtsZ by honokiol. **(B)** Time-dependent polymerization profiles of *S. aureus* FtsZ in the absence and presence of honokiol at a concentration ranging from 1 to 4 μg/mL. **(C,D)** Transmission electron micrographs of FtsZ polymers in the absence **(C)** and in the presence **(D)** of 4 μg/mL of honokiol (Scale Bar is 500 nm). **(E)** The CD spectra of FtsZ in the absence and presence of 1 or 4 μg/mL honokiol. **(F,G)** The effect of honokiol on Z-ring formation in *B. subtilis* (Scale bar is 10 μm). The bacterial cells were grown in the absence **(F)** or in the presence of 4 μg/mL of honokiol **(G)**. Comparison of the the percentage of FtsZ rings or ill-formed dots distributions in the *B. subtilis* cells treated by 1%(v/v) DMSO and the 4 μg/mL of honokiol **(H)**.

Recent studies have pointed out that the observed cell division inhibition induced by antibacterial compounds may be attributed to their ability to inhibit the GTPase activity of FtsZ (Margalit et al., 2004; Mathew et al., 2013; Sun et al., 2017b). In this study, we conducted investigations using FtsZ protein expressed from *S. aureus* to assess the effect of honokiol on FtsZ GTPase activity by applying the experimental conditions established in our previous work (Fang et al., 2019b). The results (Figure 5A) demonstrate that FtsZ GTPase activity is suppressed by honokiol in a dose-dependent manner. Notably, honokiol at 1 μg/mL, exhibited a 15% inhibition. However, at higher concentrations, such as at 2 μg/mL, 4 μg/mL and 8 μg/mL, honokiol resulted in the inhibition of 40, 60, and 75%, respectively (refer to Figure 5A). These results suggest that the inhibition of FtsZ GTPase activity by honokiol in *S. aureus* may be one of the possible mechanisms impeding bacterial growth. To further explore the effect of honokiol on the FtsZ protein activity, the dynamic polymerization of FtsZ treated with honokiol was performed. The light scattering assays were used for the investigation. Figure 5B illustrates the time-dependent polymerization patterns of FtsZ with and without using honokiol from 1 to 4 μg/mL. The results indicate that honokiol enhances FtsZ polymerization in a concentration-dependent manner, in accord with the characteristics observed in the reported FtsZ-targeting compounds (Andreu et al., 2010; Kaul et al., 2012; Kelley et al., 2012; Sun et al., 2017a). To validate the specificity of honokiol, 5 μg/mL methicillin, a non-FtsZ-targeting antibiotic, were used as a negative control, revealing no observable effects on FtsZ polymerization under the same conditions.

Additionally, we visualized the effect of honokiol on FtsZ polymerization through transmission electron microscopy (TEM). Notably, applying 4 μg/mL honokiol, both the size of FtsZ polymers and the bundling of FtsZ protofilaments exhibited a significant increase (Figures 5C,D). Collectively, these results may support that the antibacterial effect of honokiol most likely is attributed to the influence of honokiol on both GTPase activity inhibition and FtsZ polymerization enhancement. In addition to GTPase assays and FtsZ polymerization, the effect of honokiol on the secondary structure of FtsZ were investigated by monitoring changes in the far-UV circular dichroism (CD) spectrum of FtsZ (Figure 5E). The data revealed that honokiol could significantly alter the secondary structure of FtsZ. By analyzing the CD spectra according to Yang’s reference, it reveals that the secondary structure of FtsZ consists of approximately 30.9% α-helices, 21.5% β-sheets, and 47.6% other structures. When treated with 4 μg/mL honokiol, the percentage of α-helices increased to approximately 38.8%, while the percentage of β-sheets decreased to 13.9%. These structural changes induced by honokiol may disrupt the proper function of FtsZ.

### 2.7 The effect of honokiol on Z-ring formation

To understand further whether honokiol interacted with FtsZ protein in bacterial cells, the assembly of Z-ring in *B. subtilis* cells was investigated. Bacteria were subjected to DMSO (as a solvent control) or honokiol, and their fluorescence was observed with microscope. In the absence of honokiol, fluorescent spots indicating Z-rings were observed at the midpoint of the cells, and the percentages of Z-rings or ill-formed dots are 83.3 and 16.7%, respectively, (Figures 5F,H). However, for honokiol-treated bacteria, most of midcell spots were disappeared, only 10.7% Z-ring were observed (Figures 5G,H). On the other hand, most of FtsZ distribution appeared as scattered, discrete spots along the elongated cell, suggesting a mis-localization of FtsZ protein (Figure 5G; Supplementary Figure S3). Since honokiol promotes FtsZ polymerization, it is possible that the scattered and punctate fluorescence spots in the treated bacteria represent multiple, non-functional FtsZ polymer structures. The presence of such disorganized FtsZ within elongated bacterial cells is a characteristic of agents that interfere FtsZ polymerization (Hurley et al., 2016).

### 2.8 Honokiol has little impact on the mammalian tubulin

**Figure 6:**
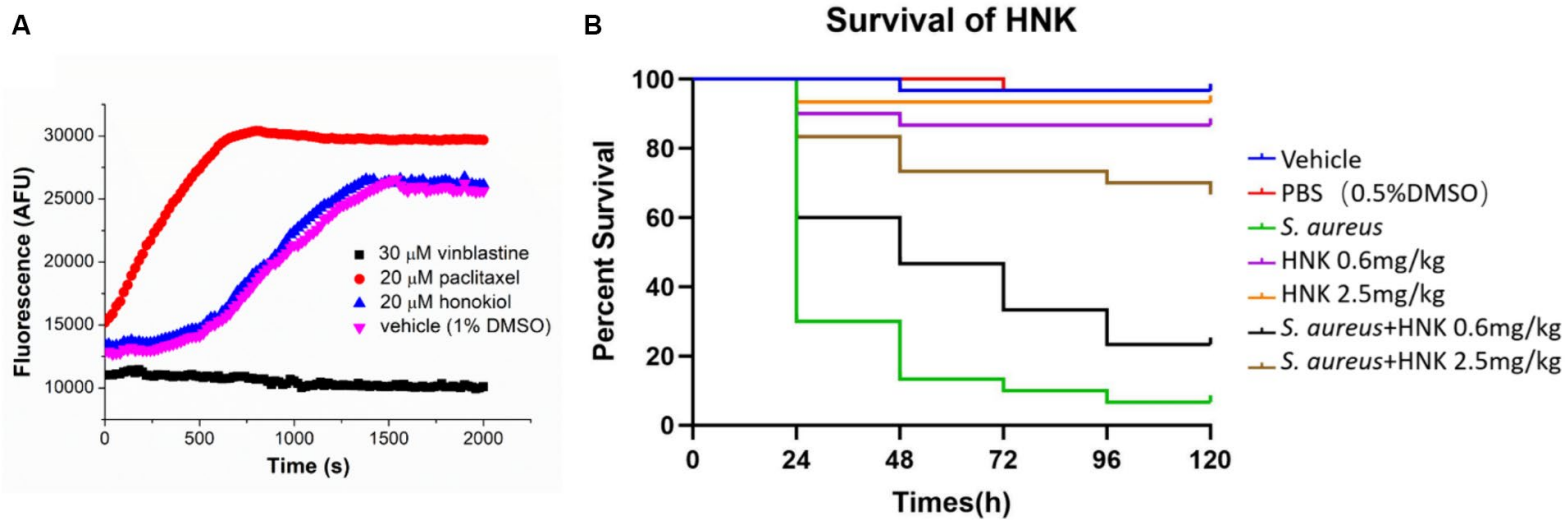
**(A)** Effect of honokiol, binblastin and paclitaxel on the polymerization of mammalian tubulin. **(B)** Evaluation of the toxicity and antibacterial activity of honokiol *in vivo* via using the *G. mellonella* larvae models (HNK: honokiol).

As tubulin closely parallels bacterial FtsZ in mammalian systems, we also assessed the potential influence of honokiol on mammalian tubulin. The presence of the tubulin inhibitor vinblastine (30 μM) caused a complete suppression of mammalian tubulin polymerization. Conversely, in the presence of paclitaxel (20 μM), a known enhancer of polymerization, a markedly increase in fluorescence intensity was observed. Notably, when applying honokiol at 20 μM, the results were found comparable with those of controls involving mammalian tubulin treated with 1% DMSO. The results suggest that honokiol neither stimulates nor inhibits tubulin polymerization (Figure 6A).

### 2.9 *In vivo* antibacterial activity of honokiol

*Galleria mellonella* larvae are widely utilized as a model for evaluating the *in vivo* toxicity and effectiveness of antibacterial drugs in non-mammalian systems (Tsai et al., 2016; Allegra et al., 2018). Our results demonstrated that the survival rates of larvae injected with 0.6 mg/kg and 2.5 mg/kg honokiol after 120 h were 89.7 and 96.6%, respectively (Figure 6B). There were no statistically significant differences compared to the vehicle and PBS group (*P*_1_ = 0.1577, *P*_2_ = 0.5480). The results may indicate that honokiol may have no or low toxicity to the larvae and it may not cause apparent harm. In contrast, the survival rate of *S. aureus* infected larvae plummeted to 6.7% after 120 h, while the honokiol-treated larvae exhibited a significantly increased survival rate at the same time point. The survival rates of the infected larvae after 120 h was 24.1% (*p* = 0.0103) and 69.0% (*p* < 0.0001) when they were treated with 0.6 mg/kg and 2.5 mg/kg honokiol, respectively (Figure 6B). This substantial difference not only shows statistically significant, but also indicates the effective rescue of larvae from *S. aureus* infection by honokiol. The results obtained from larval model may support a non-toxic profile of honokiol.

### 2.10 Proposed binding mode of honokiol in FtsZ

**Figure 7:**
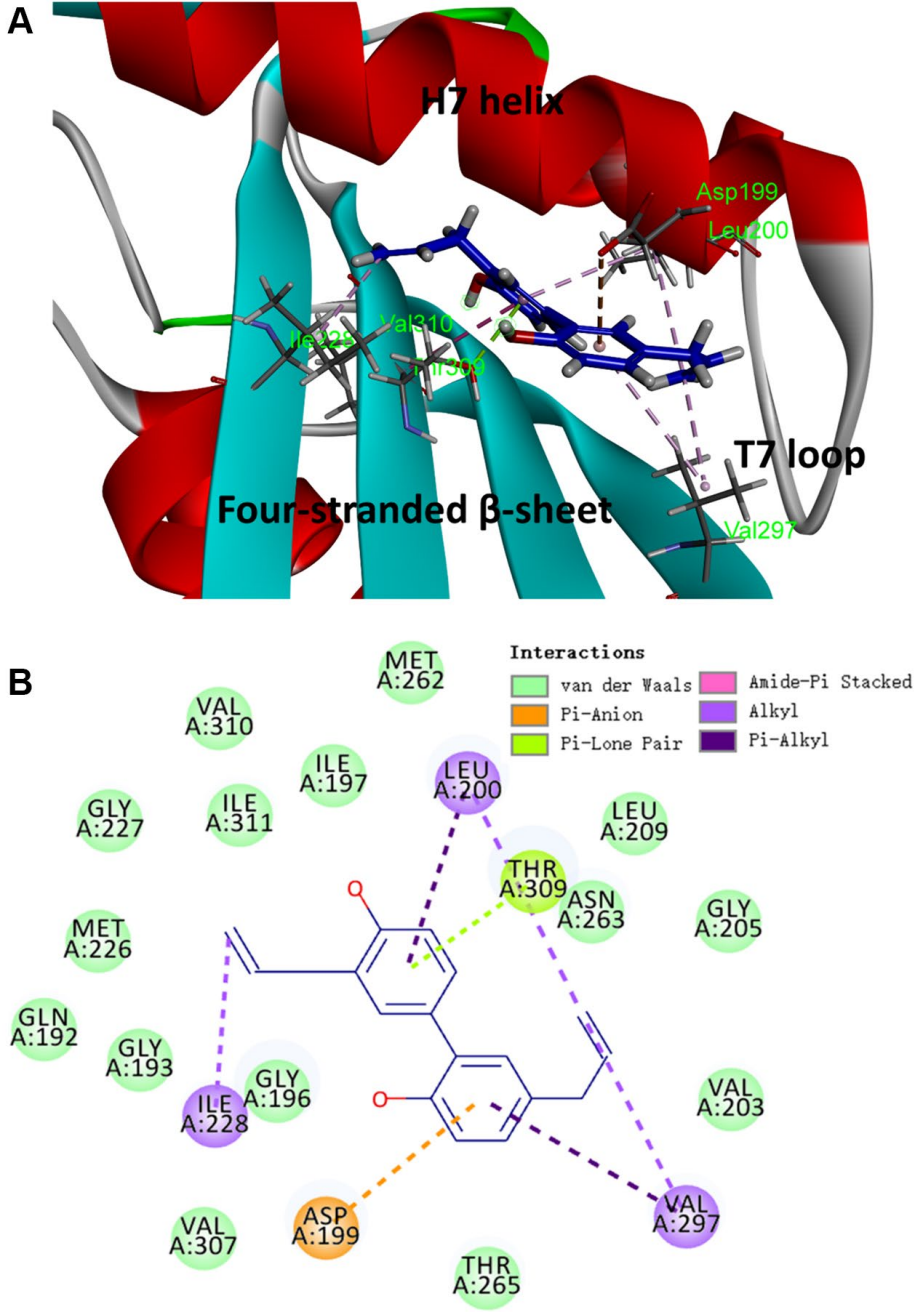
The proposed binding mode of honokiol in *S. aureus* FtsZ (PDB ID: 4DXD). **(A)** Honokiol in the interdomain cleft of FtsZ. **(B)** Predicted interaction between honokiol and amino acids of FtsZ.

Based on the results obtained from the aforementioned biological assays, honokiol may potentially interact with FtsZ. Thus, molecular modeling study was performed to predict the potential binding site of FtsZ protein. The optimal docking pose suggests that honokiol likely binds to an interdomain cleft in the C-terminal region. This narrow cleft is formed by T7-loop, H7-helix and the four-stranded β-sheet, as illustrated in Figure 7A. Further insights into the molecular interactions are revealed in the 2D ligand interaction diaGram (Figure 7B), illustrating the predicted interactions between honokiol and FtsZ residues. Hydrophobic interactions play a significant role in the binding of honokiol with the key residues of FtsZ including Asp 199, Leu 200, Ile 228, Val 297 and Thr 309. Notably, Pi-Lone Pair and Amide-Pi Stacked interactions were found between Thr 309 and the aromatic ring of honokiol. Moreover, van der Waals forces contribute to the interaction between honokiol and amino acids, such as Val 310 and Gln 192, situated around the binding pocket. The molecular interaction analysis provides the possibly molecular interaction information for honokiol binding to FtsZ protein. The present study may give valuable insights into new drug development targeting FtsZ protein for antibacterial therapy.

## 3 Materials and methods

### 3.1 Antimicrobial susceptibility assay

The *B. subtilis* strain examined in this assay was sourced from our internal collection, while additional strains were obtained from the American Type Culture Collection (ATCC, United States). Antimicrobial susceptibility tests performed were following the broth micro-dilution methods in 96-well microplates outlined in the Clinical and Laboratory Standards Institute (CLSI) guidelines (Wikler et al., 2009). For *S. aureus* strains, Cation-adjusted Mueller Hinton broth (CAMHB) assays were utilized; for *E. faecium* and *E. faecalis* strains, Brain Heart Infusion broth (BHI) assays were employed; Mueller Hinton broth (MHB) assays were used for other strains. The bacteria stored in glycerol stocks were firstly streaked onto the Luria-Bertani (LB) agar plates and then were incubated at 37°C for overnight. Then, the single colony from agar plate was inoculated in MHB (5 mL) or CAMHB (5 mL) or BHI (5 mL) and followed was cultured at 37°C for overnight with agitation at 250 rpm. The overnight-cultured cells were diluted tenfold into fresh medium and then were incubated with shaking. After shaking for 2 h, the absorbance [at 600 nm (OD_600_)] of the cell culture was measured. Then, the concentration of the cell culture was adjusted to 5 × 10^6^ CFU/mL. Subsequently, the cells (10 μL) were transferred to a 96-well microplate for measurement. The compounds to be tested were dissolved in DMSO. A two-fold serial dilution was performed for the DMSO solution. The concentration of DMSO in each well was fixed at 1%. Following a plate incubation at 37°C for 18 h, the OD_600_ values were recorded for each well with microplate reader (Bio-rad). All experiments were performed in triplicate. The minimum inhibitory concentration (MIC) was defined as the lowest compound concentration at which bacterial growth was inhibited by ≥90%.

### 3.2 Time-killing curve assay

*S. aureus* ATCC 29213 or *B. subtilis* 168 cultures underwent intentional dilution to achieve an approximate concentration of 10^5^ CFU/mL in CAMHB or MHB, respectively. The dilution process involved incorporating various concentrations of honokiol. Subsequently, the cultures were placed in a 37°C incubator with continuous shaking. At predetermined intervals, the sample (100 μL) was taken for serial dilution in 900 μL CAMHB or MHB. Following this, 100 μL aliquots from three dilutions were carefully spread onto Mueller Hinton agar plates. After a 24-h incubation period at 37°C, the plates were examined to determine accurate cell counts (CFU/mL). All experiments were performed in triplicate.

### 3.3 Synergistic effect of honokiol with β-lactam antibiotics or PMNB

Synergistic effects of honokiol with β-lactam antibiotics or PMNB were examined with checkerboard assays (Tsubery et al., 2000). Honokiol solution was diluted in 2-fold serial dilutions across the microplate (96 wells), while β-lactam antibiotics or PMNB were similarly diluted down the 96-well assay plate. Two columns of the 96-well assay plate were reserved for untreated cells. Then, the bacterial suspensions at 5 × 10^5^ CFU/mL were added to each well of the assay plate to 100 μL. All experiments were performed in triplicate. The assay plates were then incubated at 37°C. After incubated for 18 h, a microplate reader was used to measure the OD_600_ value for the wells of the 96-well assay plate.

### 3.4 Visualization of bacterial morphology, bacterial membrane and Z-ring

The morphology of *B. subtilis* cells was examined with an Olympus FSX100 microscope. Log-phase *B. subtilis* cells were diluted to the OD_600_ of 0.1 and subjected to incubation, with or without the presence of honoiol, at 37°C for 4 h. Following incubation, the bacterial cells were harvested and re-suspended in a PBS buffer containing 0.25% agarose. A volume of 10 μL from the sample mixtures was then applied to the microscopic slide pre-treated with 0.1% (w/v) poly-L-lysine. For the purpose of membrane staining, *B. subtilis* cells were subsequent incubated with a concentration of 1.6 μM of FM 4–64 for an additional half-hour at a temperature of 37°C, with no shaking involved. Post-incubation, the cells were harvested and then re-suspended in a 100 μL solution of PBS buffer, which included 0.25% agarose. Subsequently, a volume of 10 μL from this cell suspension was depositied onto a microscope slide that had been pre-coated with 0.1% (w/v) poly-L-lysine. The morphology of the bacterial cells was subsequently examined under a light phase-contrast microscope using an Olympus Bio Imaging Navigator FSX 100 microscope. For the visualization of Z-ring, a culture of *B. subtilis* harboring an IPTG-inducible plasmid for the overexpression of GFP-tagged FtsZ was cultivated in LB medium supplemented with 30 μg/mL chloramphenicol. Following an overnight incubation, a portion of the culture was serially diluted to 1% in fresh LB medium supplemented with 4 μg/mL of honokiol and 40 μM of IPTG. The cells were then incubated for an additional 4 h at 37°C. Subsequently, the cells were fixed, collected, and re-suspended in PBS buffer containing 0.25% agarose. A volume of 10 μL of the cell suspension was added to a microscope slide pre-coated with 0.1% (w/v) poly-L-lysine and examined using a fluorescence microscope at 60× oil immersion objective with a standard FITC filter cube. The images were obtained using an Olympus Bio Imaging Navigator FSX 100 microscope system.

### 3.5 GTPase activity test

The GTPase activity of *S. aureus* FtsZ was assessed following established protocols outlined in our prior research (Fang et al., 2019b). A phosphate assay kit was employed for the evaluation, conducted in 96-well microplates based on a previously described methodology. In this experiment, the FtsZ proteins (4 μM) were incubated with honokiol at various concentrations in Tris buffer (20 mM, pH 7.4) for 10 min at room temperature. To prevent compound aggregation, 0.01% Triton X-100 was included. Following this, 5 mM MgCl_2_ and 200 mM KCl were added to the reaction mixture. The reactions were initiated by introducing 500 mM GTP and allowed to incubate at 37°C. All experiments were performed in triplicate. After a 30-min incubation period, the reaction was terminated with the addition of Cytophos (100 μL). Then, the sample was further incubated for 10-min. The quantification of inorganic phosphate was conducted with a microplate reader to record the absorbance at 650 nm.

### 3.6 Effects of honokiol on the FtsZ polymerization

For scattering assays, a fluorescence spectrometer was employed to detect FtsZ polymerization under the condition reported previously (Fang et al., 2019b). FtsZ concentration at 7.5 μM in a MOPS buffer (50 mM, pH 6.5, supplemented with 0.01% Triton X-100 to prevent compound aggregation) and honokiol at various concentrations were loaded into a fluorometer cuvette. By sequential additions of 50 mM KCl, 10 mM MgCl_2_ and 1 mM GTP, the polymerization reaction was initiated and then was monitored for 2000 s. The experiments were performed in triplicate. For the TEM analysis, 12 μM *S. aureus* FtsZ were incubated in the absence and in the presence of honokiol (4 μg/mL) in MOPS buffer (50 mM, pH 6.5) under room temperature conditions. After incubation for 10 min, the reaction mixtures were supplemented with 50 mM KCl, 5 mM MgCl_2_ and 1 mM GTP, followed by a subsequent incubation at 37°C for 15 min. Next, the resulting sample mixtures (10 μL) were deposited onto a 400-mesh glow-discharged Formvar carbon-coated copper grid for a 10-min duration. The grids were then negatively stained using 0.5% phosphotungstic acid (PTA, 10 μL) for 30 s, air-dried, and subjected to observation.

### 3.7 Effect of honokiol on the secondary structural changes of FtsZ

*Sa*FtsZ (10 μM) was allowed to react for 30 min at 25°C with either no addition or with various concentrations of honokiol (1 and 4 μg/mL) in a 20 mM Tris buffer (pH 7.4), which included 0.01% Triton X-100 to prevent the compound aggregation. The far-UV circular dichroism (CD) spectrum was recorded over the wavelength range of 200–250 nm using a JASCO J-810 spectropolarimeter that was fitted with a temperature controller and a 0.1 cm path length quartz cuvette. Each spectrum was an average of five scans. The CD spectra were analyzed for deconvolution and statistical purposes using Jasco analysis software and Origin 8.0 software, respectively.

### 3.8 Impact of honokiol on eukaryotic tubulin polymerization

The eukaryotic tubulin polymerization in the presence of honokiol was studied by employing a tubulin polymerization assay kit (BK011P, Cytoskeleton, Inc.) with fluorescence microscopy. The process of polymerization was observed by tracking the increase in fluorescence intensity from a fluorescent marker, 4′,6-diamidino-2-phenylindole (DAPI), which was integrated into the microtubules. The concentration of porcine brain tubulin used in the assay was 2 μg/mL. As control references, paclitaxel (20 μM) and vinblastine (30 μM), which were known to either promote or inhibit tubulin polymerization, respectively, were also included. The test compound, honokiol, was administered at a concentration of 20 μM, which equated to 5.3 μg/mL in a final solution of 1% DMSO. All experiments were performed in triplicate. The fluorescence readings were obtained using a PolarStar Optima microplate reader, with the excitation wavelength set at 360 nm and the emission wavelength at 450 nm. The collected data were managed using Microsoft Excel and further analyzed with Origin analysis software.

### 3.9 Impact of honokiol on *Staphylococcus aureus* infection *Galleria mellonella* larvae model

*G. mellonella* larvae weighing around 0.25 g were selected randomly. Then, the honokiol solution (20 μL) was injected per larvae with a microinjector (HAMILTON, Swiss). Three independent experimental groups (10 larvae per group) were conducted. The experimental groups were tested under the following conditions: vehicle, PBS (0.5% DMSO), infected group (*S. aureus* ATCC 29213 suspension was injected and the final concentration was 0.5 McFarland), the compound group (injected with honokiol 0.6 mg/kg or 2.5 mg/kg) and the treatment group (injected with honokiol 0.6 mg/kg or 2.5 mg/kg after 2 h *S. aureus* injection). All experiments were performed in triplicate. Following injection, the larvae were cultured in a dark room at a temperature of 37°C. Subsequently, the number of viable larvae in each experimental group was counted at 24-h intervals for a duration of 120 h. The survival rate was then computed and graphically represented (Peng et al., 2023). Statistical analysis and graphical representation of the experimental outcomes were conducted with GraphPad Prism 8.0 software (GraphPad Software, San Diego, California, United States). The experimental data were presented as mean ± standard deviation (x ± s). Significance assessment was performed using the Log-rank test; statistical significance was denoted by *p* < 0.05, with *p < 0.05, **p < 0.01, and ***p < 0.001 indicating varying degrees of significance.

### 3.10 Molecular modeling analysis

For our molecular modeling investigation, we utilized the CDocker program within Discovery Studio 2016. An X-ray crystal structure of *S. aureus* FtsZ, sourced from the RCSB Protein Data Bank (PDB entry: 4DXD) (Tan et al., 2012), was employed as the foundation. Elimination of water molecules and co-crystal ligands, as well as protein preparation for docking, was carried out using an automated protocol in Discovery Studio. The binding sites of FtsZ were defined from PDB record. The molecular structure of honokiol was hand-drawn and converted into a three-dimensional format through the Discovery Studio molecule editor. The automated docking study was then executed with DS-CDocker protocol. CDOCKER is a software application that employs the CHARMM force field for molecular docking, utilizing a rigid receptor model. Random conformations of honokiol were spawned through high-temperature molecular dynamics simulation, involving 1,000 dynamic steps conducted at 1,000 K. These diverse conformations of honokiol were subsequently docked into the binding site, with their orientations refined through simulated annealing molecular dynamics consisting of 2,000 steps heated to 700 K followed by 5,000 steps to cool down to 300 K. The energy values of the final docking poses were computed and subsequently ordered based on their respective CDOCKER scores. The resultant highest-scoring pose underwent thorough visual inspection for a detailed analysis of molecular interactions.

## 4 Discussion and conclusion

In recent years, increasing evidence suggest that honokiol possesses a wide range of pharmacological activities (Rauf et al., 2021). For instance, honokiol inhibits the growth and spread of tumor cells through various pathways. Honokiol exerts anti-tumor effects through multiple mechanisms, including induction of apoptosis, inhibition of cell proliferation, and suppression of angiogenesis. Honokiol has shown promising results in preclinical studies against various cancers, including breast, lung, prostate, and pancreatic cancer (Cen et al., 2018; Ong et al., 2019; Zhang et al., 2019).

Honokiol also possesses strong antioxidant properties, scavenging free radicals and reducing oxidative stress (Rauf et al., 2021). Its antioxidant activity has been linked to protection against neurodegenerative diseases, cardiovascular disorders, and age-related cognitive decline (Wang et al., 2011; Talarek et al., 2017; Wang et al., 2022). Moreover, honokiol exhibits potent anti-inflammatory effects by suppressing the production of pro-inflammatory cytokines and inhibiting NF-κB signaling pathways. Studies have demonstrated its efficacy in ameliorating inflammatory conditions such as arthritis, colitis, and dermatitis (Tse et al., 2005; Debsharma et al., 2023).

Furthermore, in a 30-day clinical trial with 40 participants, no significant side effects were found from daily doses of 11.9 mg of honokiol (Campus et al., 2011). Given the safety and pharmacological activity of honokiol, it shows promise for further development as a natural compound-based drug (Sarrica et al., 2018).

Recently, the antibacterial activity of honokiol has been reported (Rauf et al., 2021). Honokiol at 10 μg/mL was found to inhibit biofilm formation of MRSA 41573 and, at 50 μg/mL, it disrupted the mature biofilm. The results of RT-PCR analysis suggests that its potential mechanism of action may involve the inhibition of sarA, cidA, and icaA, as well as eDNA release, and the expression of PIA (Li et al., 2016; Qiao et al., 2016). Despite honokiol is reported to inhibit the growth of *Actinobacillus actinomycetemcomitans, Porphyromonas gingivalis,* and *Prevotella intermedia* with a MIC value of 25 μg/mL (Chang et al., 1998; Ho et al., 2001), the inhibition mechanism of honokiol is still unclear at present. By using computer-aided simulation, Liu et al. suggested that honokiol could bind to the PC190723 binding site of FtsZ protein. Biological tests also found that honokiol could inhibit FtsZ polymerization at a high concentration (100 μg/mL). It also exhibited antibacterial activity against three strains of *S. aureus* tested with MIC values of 8–16 μg/mL (Liu et al., 2014). However, in the reported study, neither the effects of honokiol on FtsZ GTPase activity, nor its inhibitory effects on bacterial cell division were investigated. Additionally, Triton X-100 was not added in the assessment of honokiol’s inhibitory effect on FtsZ. Since some small molecules may inhibit FtsZ polymerization through compound aggregation, the use of Triton X-100 in the assays is required to exclude false positive results when testing the effect of compounds on FtsZ polymerization (Anderson et al., 2012).

In the present study, we revealed that honokiol effectively enhanced FtsZ protein assembly and inhibited GTPase activity of FtsZ, while the compound showed no any observable effects on tubulin polymerization. Moreover, honokiol was found inhibited bacterial cell division. In the antibacterial tests, honokiol displayed potent antibacterial effects against Gram-positive bacteria including VREF and MRSA. In addition, honokiol restored MRSA susceptibility to the β-lactam antibiotics tested. Furthermore, the growth of Gram-negative bacteria was inhibited when honokiol was combined with PMBN. The anti-infective potential of honokiol against bacteria was further validated with the use of *G. mellonella* larvae as an *in vivo* model. The notable features of honokiol position it as a promising candidate for further structural modifications. This study is intended to bolster its pharmacological activities, refine pharmacokinetic profiles, and ultimately pave the way for developing potent antibacterial agents against drug-resistant bacterial infections.

## Data availability statement

The original contributions presented in the study are included in the article/Supplementary material, further inquiries can be directed to the corresponding authors.

## Ethics statement

The manuscript presents research on animals that do not require ethical approval for their study.

## Author contributions

NS: Conceptualization, Data curation, Funding acquisition, Investigation, Project administration, Resources, Supervision, Writing – original draft, Writing – review & editing. ZZ: Data curation, Investigation, Methodology, Software, Writing – original draft. TX: Methodology, Writing – original draft. XD: Methodology, Writing – original draft. TH: Software, Writing – original draft. WD: Software, Writing – original draft. SF: Software, Writing – original draft. SC: Software, Writing – original draft. W-LW: Conceptualization, Funding acquisition, Supervision, Writing – review & editing. WY: Conceptualization, Funding acquisition, Project administration, Resources, Supervision, Writing – review & editing.
