# Supplementary material for: The study of honokiol as a natural product-based antimicrobial agent and its potential interaction with FtsZ protein
Source: Front Microbiol. 2024 Jul 22;15:1361508. doi: 10.3389/fmicb.2024.1361508 (PMC11298477; doi:10.3389/fmicb.2024.1361508)
Supplement: Supplementary file 1 [file Table_1.DOCX]

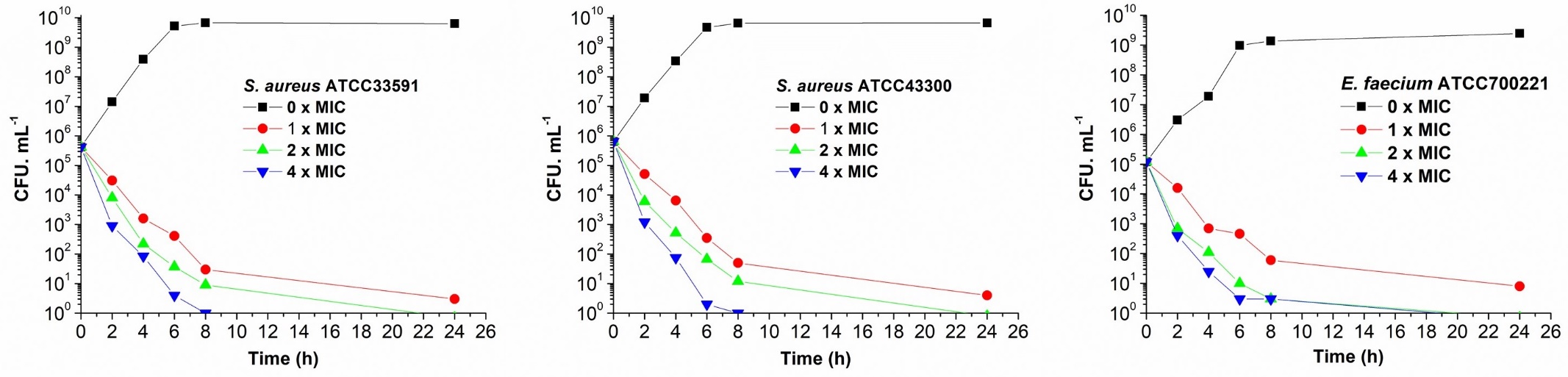


Figure S1. Time-killing curve of honokiol against drug-resistant *S. aureus* ATCC 33591 and ATCC 43300, and *E. faecium* ATCC 700221.
